# Monthly Summary

**Published:** 1887-01

**Authors:** 


### Monthly Summary.

---

FATAL RESULT OF PEROXIDE OF HYDROGEN INJECTIONS. --A Norwegian surgeon, Dr. Laache, has reported a case in which a fatal result followed an injection of peroxide of hydrogen into the pleural cavity. The patient was a man 28 years of age, who had had a portion of the ninth and tenth ribs resected, for empyema. The operation had been successful and the wound progressed favorably, and two months after the operation there remained only a fistula about an inch and a half long. In order to hasten the healing of this, hypodermic injections of a 3 per cent. solution of peroxide of hydrogen were resorted to, as this method of treatment had given very satisfactory results in two somewhat similar cases in which it had been tried. Six injections, each containing 0.8 cubic centimetre of the solution, were administered without any particular effect. At the seventh however, the patient complained of pain and faintness, the pulse failed, the respiration became oppressed, clonic contractions occurred in the right arm, the head turned to the left, the jaws became tightly set, the face became cyanotic, and the patient died in ten minutes. The necropsy, which was not made till forty-eight hours after death, revealed nothing very striking. The heart was dilated, and contained liquid blood without air bubbles ; some ecchymoses were found in the parts of the left lung, which were adherent to the chest walls. In the fourth ventricle of the brain an ecchymosis the size of a pin's head was seen. A number of air bubbles were present in the blood of the hepatic veins, and some were seen on cutting into the spleen and kidneys. The cause of death was therefore by no means clear. Dr. Laache suggests that it may have been the small extravasation in the fourth ventricle or shock. Dr. Wulfsberg, who took part in the discussion on the case at the Christiania Medical Society, thought it was probably due to the introduction of the drug into the circulation. He thought that the strength of the blood current might have carried the peroxide, if introduced into a vein, through the right heart and lungs almost unchanged, but that afterwards more oxygen might have been disengaged than the blood could absorb, and bubbles were thus produced, which may have been the immediate cause of death. Where animals have been subjected to injections of air into the veins bubbles are not always found in the blood. In these cases Dr. Wulfsberg thinks that death has been due to paralysis of the heart, for when examinations have been made immediately after death bubbles have been found in the coronary arteries. As so long a time had elapsed before the necropsy in Dr. Laache's case, these might have been absorbed.—*London Lancet.*

---

HOW TO BECOME A GOOD DENTIST.—*Dr. B. H. Teague.*—After two years of close application under a conscientious and painstaking preceptor, the student is then ready to enter the dental department of a university. The intervening time between the winter sessions should again be spent under the supervision of the preceptor; and after graduation a year with him as an assistant, will be of much benefit to the newly fledged dental doctor.

It has been my observation that the graduate who has not availed himself of the benefits of a private tutor, may be skillful after a manner with cohesive gold, may know little of non-cohesive gold, is sure to dislike amalgam and other plastics, and is a very indifferent plate-workman—even with rubber. He is a bungler at such fine prosthetic dentistry as pivoting, cap-crowning, and bridge-work. In fact, is almost an ignoramus, practically, in all that relates to artistic metal work. Often I have had said to me, "Why, Doctor, they never taught me *that* at college." The colleges cannot demonstrate everything. In all probability, the different phases of practice are described in the lectures; but the most interesting and special practice is not practically taught the student, unless occasionally by some dentist invited to give a clinic. The patients who are served at the college infirmaries are generally poor, and do not demand anything more than either simple operative or mechanical dentistry. The scope is therefore limited as compared with the daily routine of a busy dentist. A graduate who has received instruction only within the walls of a college should place himself with a well-established dentist as an assistant. The time spent will not be thrown away. A practical knowledge will be thus gained to help him over many of the rough and troublesome obstacles in practice. He will better satisfy himself and more efficiently serve his patrons, and receive the greater pecuniary return from future practice.—*Ex.*

---

IN FILLING PROXIMAL CAVITIES, should the teeth be wedged previous to filling, or cut apart and permanent separations made? Which plan will be likely to result in the most good, both as to durability and comfort to patient? I should say as a general rule it is best to wedge the teeth rather than cut spaces, and yet many times the latter seems the best plan to adopt. I consider this one of the most difficult questions to decide in dental practice, and one requiring much thought and good judgment. The shape of the teeth, the way they antagonize, the tendency for caries to recur, the character and shape of the gums, the age of the patient, and care taken of the teeth, must all be taken into account. As a general rule, teeth that are wide at the grinding surfaces and small at the necks should not be cut apart, because the wide space at the gums interferes with mastication. If the gums and alveolus are heavy, there is apt to be a pocket formed, which adds greatly to the chance of caries recurring, besides giving discomfort to the patient in chewing, if permanent spaces are made through to the gums.

If the occlusion is such that when spaces are cut the teeth will soon crowd together again, cutting apart is generally bad practice; or if the patient is quite young, so that the teeth scarcely occupy their natural positions, cutting apart is hardly justifiable. On the other hand, where there is much tendency to caries with a lack of care on the part of the patient, contouring is not good practice, because the time and exhaustive labor required for such operations are almost sure to be rewarded by a quick recurrence of caries. Permanent separating is then almost the only thing left. So that no special rule can be given, but each case must be left to the careful study and good judgment of the operator. Cleanliness is also very important Till we get patients to doing their part in taking proper care of the teeth, it is of but little use to try to make permanent operations, This is especially true with regard to children. I have repeatedly sent children home and set another time for them to come on purpose to examine and show them where they fail in this particular. It is said that the pulp-capping practice has changed. I have seen no reason for changing my practice in this particular. I cap pulps quite as frequently as I ever did, have quite as much faith in it, and believe any practitioner who does not cap pulps falls far short of his duty.—*Dr. J. N. Crouse.*

---

PERSONAL CLEANLINESS AND UNPLEASANT ODORS, AND DISEASE.—Sometime since, referring to personal offensive odors, we laid stress on keeping the entire body clean, of having good digestion, and of avoiding foods tending to taint the perspiration. We would like to extend those remarks.

And to the last first. A dentist certainly has no right to eat cabbage and onions and other offensively odoriferous foods. And if these are to be avoided because of their offense to others, we should seek those things which produce a pleasant aroma. A dish of nice strawberries or pineapple, a handful of wintergreen berries and an occasional piece of sassafras, spikenard, ginseng, and many other vegetables, fruits, and aromatics, impart to the breath and to the insensible perspiration an agreeable aroma that should be prized. If *something* must be chewed, instead of giving your patients the abominable stench of nasty tobacco, take tolu, tumerac, or almost any of the balsams.

But be as careful as we may of what we eat, or use aromatics as much as we may, if we give the stomach more food than it can digest, there will be a decay of part of it which will send out defection and disease into all parts of the system, and surround the body with the odor of death. Most of us eat too much, and our food is too rich and concentrated. We are gormandizers, and it is no wonder so much is rejected by the delicate digestive follicles; and that the rejected matter sends out the offensive smell of putrefaction. Yes, and more than this, the blood made by such a mass as is in the stomach, cannot be healthy. Therefore, as one organ after another receives the illy prepared blood, with effete matter circulating in it, we have impaired function and a diseased condition of the organs. The organ that is the weakest becomes the most affected, and then we name the disease after that organ. The poisonous matter thus taken up by the lymphatics is also brought to the skin to produce pimples and blotches and cutaneous affections; on its way it clogs the circulation, producing what we call rheumatism and other painful disorders. Thus we have from undigested food disorganized and disorganizing matter. O, my; what foolish creatures we are! How much easier it is to prevent disease than to cure it. We sometimes think it is a sin to be sick; we would be almost sure of it if we were not occasionally sick ourself. It is certainly often our own fault.

But though we may be judicious in the quantity and quality of our food, there will be offensive material and gases thrown to the surface through the pores of the skin. This is nature's outlet for them; and if by its healthy condition, and by our intelligent management, it is able to do its work well and promptly, these exudations are easily managed.

Of course we all know that we are covered with scales, that is, if we are not cleanly. It is like the dandruff of the scalp—with those who are too lazy to remove it. Those who properly and frequently clean their skin have few of these scales of dead scarf skin; the surface is almost as soft and transparent and lovely as a child's.

The true skin under this is not a simple protection. Running up through it from the suderiforous glands in the under layer are spiral, hollow tubes, which with their glands are from an eighth to a half inch long according to the thickness of the skin, and from a tenth to a thirtieth of an inch in diameter. There are more than two million of them, continually sending steam and gas to the surface in the form of sweat, and with this, effete, gummy material that is deposited on the surface. If this matter is not frequently removed it closes the pores, and is a stench. No one can disguise it, and if left long will proclaim its presence loudly, at least to the olfactories of our patients. On some parts of the body, as the palms of the hands and the soles of the feet, there are 2800 of these pores to the square inch. If the two million five hundred thousand in the skin of a grown person were put length to length they would reach more than nine miles! But in this form they would not do the good they accomplish in their short lengths. Each with its mouth open breathes in the pure oxygen of the air, and breathes out the smoke of the internal fire in the form of carbon; and with this carbon spits out the effete matter sent into their throats from the venous blood. We are confident the importance of the healthy functions of these outlets are not generally appreciated. Why, they are as indispensable as the proper action of the liver or the kidneys.

If by a sudden chill the mouths of these tubes become constricted, how quickly fever comes; and much of the enervation and laziness we sometimes feel is by their being closed with this dead, gummy matter of which we have spoken.

Jump naked into a wet sheet; have it packed tightly round you, and then quilts bound round this, till you are made a mummy; then drink copiously of cold water and go to sleep. In fifteen or twenty minutes you will begin to sweat from head to foot, and your wet sheet will become a universal poultice, Don't be in a hurry to come out; stay at least an hour and a half, and three hours won't hurt you. Now with lots of hot water and soap scour yourself. You never saw such a scum on the surface of the water you ever before washed in! Where could it all have come from? And that wet sheet; see how perfectly covered and saturated it is with offensively smelling gummy stuff? It can hardly be washed out. But before wiping yourself dash on cold water, and then rub hard and briskly till the whole skin glows. Ah, how good you feel now. You want to run and skip and jump. Well, this is nature's way of telling you to do it, and it is nature's proof of the sanitary good of the operation. Try it, you that have an offensive odor, and see how a few such renovations will make you as sweet as a summer's rose. Try it, you that feel dull and lazy, languid and drowsy, enervated and "billious." and see how quickly it will make you young again. Try it, you old, worthless, rheumatic, gouty, skin bound grumblers and grunters, and see how a few will straighten you out. But those, dirty, saturated, "highly scented" underclothes you took off when you entered the hydropathic pack—don't put them on again, take clean ones. Just weigh your flannels when they are clean and when you have worn them a week, and see the difference. This should convince anyone of the importance of frequent changes and frequent washings.—*Items of Interest.*

---

PHYSICIANS EXTRACTING TEETH—A physician writing on Mistakes in Practice, among other things says:—I never received any training in the extraction of teeth, and when a student in the office of my preceptor, I made a total failure at one time in trying to extract a tooth. That should have been a sufficent lesson to me, but it wasn't. I occasionly receive a call to extract a tooth. I do not own a pair of tooth forceps, and always send such calls to a dentist. One rainy night, about three years ago, I was prevailed upon to visit a lady for the purpose of extracting a tooth. I was told that the friends had a pair of forceps. The truth is, I haven't confidence enough in myself to extract a tooth. I failed, of course. I then furnished my horse and carriage, gratis for the friends to take her to a dentist. Every failure, however small, cannot help but tarnish a man's professional reputation. I have not attempted to extract a tooth since, and when I do, "it will be a cold day." It is true that country practitioners must extract teeth, and I believe that that branch of *minor surgery* should be taught, practically, in our medical colleges as much as any part of the student's equipment for his future duties. A little actual practice, under the guidance of an experienced operator would go a good ways in establishisg a professional reputation. —*Items of Interest.*

---

THE MOST POPULAR MEDICINES.—Last month we published a table compiled by an American writer showing the comparative proportion in which twelve of the leading medicines had been ordered in 1,000 prescriptions which had been taken at random. It was shown that quinine was ordered 238 times, opium 136, nux vomica 130, iron 123, while iodine, mercury, bismuth and bromine are altogether at 59 and 60 times. In June 1868, we published an article by Mr. W. Willmott on "Medicine," in which a somewhat similar investigation is recorded. Mr. Willmott had analysed 1, 000 prescriptions, but he did not give the details in a form which admits of exact comparison. He, however, found that quinine was far ahead of any other single medicine ordered, but classifying all remedies in their natural groups, he found mercury prominently at the top, then potash, bark, opium and iron. He found that out of the 768 simple and compound medicaments of the pharmacopœia, only 485 occured at all in these 1,000 prescriptions, while three-fourths of these were not prescribed 10 times in the 1,000.—*Chemist and Druggist.*

---

DENTISTS DAMAGED BY THE EARTHQUAKE AT CHARLESTON.—Dr. Bull lost his residence and office by fire; saved his chair and instruments. His loss is heavy. Dr. Miles's place is so badly damaged that he will have to move. Dr. O'neil suffered great loss. Dr. J. B, Patrick had his eyes severely hurt by falling plaster. Dr. Brown's damage will amount to two hundred dollars.—*Southern Dental Journal.*

